# Electromyographic Analysis of Paraspinal Muscles of Scoliosis Patients Using Machine Learning Approaches

**DOI:** 10.3390/ijerph19031177

**Published:** 2022-01-21

**Authors:** Ruixin Liang, Joanne Yip, Yunli Fan, Jason P. Y. Cheung, Kai-Tsun Michael To

**Affiliations:** 1 Laboratory for Artificial Intelligence in Design, Hong Kong Science Park, New Territories, Hong Kong 999077, China; cynthia.rx.liang@connect.polyu.hk; 2 Institute of Textiles and Clothing, The Hong Kong Polytechnic University, Hung Hom, Kowloon, Hong Kong 999077, China; 3 Department of Orthopaedics & Traumatology, LKS Faculty of Medicine, The University of Hong Kong, Pokfulam, Hong Kong 999077, China; fanyl@hku-szh.org (Y.F.); cheungjp@hku.hk (J.P.Y.C.); mikektto@hku.hk (K.-T.M.T.); 4 Department of Orthopedics, The University of Hong Kong—Shenzhen Hospital, Futian District, Shenzhen 518000, China; 5 Physiotherapy Department, The University of Hong Kong—Shenzhen Hospital, Futian District, Shenzhen 518000, China

**Keywords:** muscle activity, random forest, support vector machines, importance analysis, asymmetry

## Abstract

A large number of studies have used electromyography (EMG) to measure the paraspinal muscle activity of adolescents with idiopathic scoliosis. However, investigations on the features of these muscles are very limited even though the information is useful for evaluating the effectiveness of various types of interventions, such as scoliosis-specific exercises. The aim of this cross-sectional study is to investigate the characteristics of participants with imbalanced muscle activity and the relationships among 13 features (physical features and EMG signal value). A total of 106 participants (69% with scoliosis; 78% female; 9–30 years old) are involved in this study. Their basic profile information is obtained, and the surface EMG signals of the upper trapezius, latissimus dorsi, and erector spinae (thoracic and erector spinae) lumbar muscles are tested in the static (sitting) and dynamic (prone extension position) conditions. Then, two machine learning approaches and an importance analysis are used. About 30% of the participants in this study find that balancing their paraspinal muscle activity during sitting is challenging. The most interesting finding is that the dynamic asymmetry of the erector spinae (lumbar) group of muscles is an important (third in importance) predictor of scoliosis aside from the angle of trunk rotation and height of the subject.

## 1. Introduction

Scoliosis is a three-dimensional spinal deformity usually associated with intervertebral rotation [1]. Among the different types of scoliosis, adolescent idiopathic scoliosis (AIS) is most common and estimated to affect 2–3% of the general population [2]. Scoliosis can also affect the physical and mental health of patients [3,4,5]. During the past few decades, different factors that affect the prevalence of scoliosis have been identified, such as gender, age, skeletal maturity, body mass index, family history, and ethnicity [6,7,8,9,10,11,12]. For instance, the probability of severe AIS was 5 to 10 times more prevalent among females than males [8]. A notable characteristic of females with scoliosis was that their height was higher than that of average females at the age of 11–15 [11]. Moreover, there was a higher prevalence of scoliosis when the age was older than 15 years (after puberty) [12].

At the same time, more factors are being revealed. Recently, Chen et al. [13] suggested that imbalanced growth between the thoracic vertebral column and the sternum is also an important causative factor for the pathogenesis of scoliosis. However, these factors have not been examined concurrently in previous studies in the literature, and their correlation with scoliosis has not been ranked. This then leads to the question of whether a selected variable is really relevant and useful for conducting classifications and making predictions in scoliosis classification studies. In addition, although guidelines have been well established for good practices in controlling spinal curvature, the selection of treatment type is often subjective and based on the experience of clinicians [14]. The evaluation of treatment effectiveness mainly relies on X-rays [15]. Clinicians are now looking for more effective prognostication and evaluation methods to provide a better treatment outcome [16,17].

Electromyography (EMG) has been well used to evaluate and record the electrical activity of skeletal muscles in a number of studies [18,19,20,21]. It is also popular in studies of paraspinal muscles for scoliotic patients. Over two decades ago, Avikainen et al. [22] recorded the EMG signals of the paraspinal muscles of girls with AIS versus time and compared them with those without the condition. Their results showed that the integrated EMG signals of the left lumbar muscles are higher than those of the right lumbar muscles at the start and during the early stages of muscle contraction for those with AIS. However, they did not observe any differences in the maximal integrated EMG activity. More recently, Chwała et al. [23] examined the differences in the EMG readings of the paraspinal muscles during symmetric loading and asymmetrical stretching exercises in girls with idiopathic scoliosis. They found a significant difference in the muscle activation patterns between the two types of physiotherapy exercises. Stetkarova et al. [24] used needle EMG instead of surface EMG (sEMG) to investigate the changes in the paraspinal muscles of AIS patients, and found higher amplitudes of the motor unit potentials on the convex side of the scoliosis curve. They also conducted a muscle biopsy and found predominantly type I muscle fibers in the curve convexity. The type I muscle fibers show higher fatigue resistance and lower contractile speed as opposed to the type II muscle fibers [25]. Moreover, it is also found that AIS progresses more rapidly depending on the Cobb angle, which is significantly correlated with the increased proportion of type I fibers on the convex side of the scoliotic curve [24,26]. The changes might cause the asymmetry of muscle activity. However, these findings lead to the question of whether asymmetric paraspinal muscles cause the emergence of AIS or vice versa. The studies also have limitations. Although many have focused on female participants since a higher percentage of patients with scoliosis are female [22,23,24], nevertheless, the gender bias inhibits the generalizability of the results. In addition, most of these studies have only focused on no more than 12 participants or even as few as 10 participants [19,20,22,24]. The results again would be difficult to generalize.

In terms of the analysis method, data-driven scientific research and artificial intelligence (AI) have greatly improved clinical studies on scoliosis treatment. Electronic health records and wearable devices provide the means for researchers to develop sophisticated machine learning (ML) algorithms, which can be used to quickly advance treatment. Some researchers have even applied different ML technologies to classify spinal curvatures and facilitate early diagnosis. For example, convolutional neural networks (CNNs) are a deep learning algorithm and highly accurate (98.3%) in classifying different treatments for scoliosis based on posteroanterior radiographs [27]. Still, challenges remain as most of these approaches are only based on X-ray images. Yang et al. [28] used deep learning algorithms on 3240 images taken of the bare back of male and female patients to identify the presence of a scoliosis curvature. They found that the accuracy of the algorithms exceeds that of human diagnoses; however, the algorithms can only identify scoliosis curvatures that are more than 20 degrees. Nevertheless, their approach can directly reduce radiation exposure to X-rays of potential patients and medical costs, and is time efficient.

The aim of this study is to investigate the characteristics of participants with imbalanced muscle activity and the relationship between physical features and EMG signals. This study involves more than 100 participants, including participants with and without scoliosis of both genders. The prediction of scoliosis is made possible by using random forests and support vector machines (SVMs). Furthermore, an importance analysis is conducted by using random forests because this method is inclusive and can deal with small sample sets [29]. An importance analysis can identify important causative factors to accurately detect scoliosis. The findings can further facilitate investigations on the features of paraspinal muscles that have a key role in diagnosing AIS and increasing the likelihood of their future application for examining the pathogenesis of scoliosis and exploring the application of ML methods in scoliosis-related studies.

## 2. Materials and Methods

### 2.1. Participants

In total, 106 participants were consecutively and randomly recruited (69% scoliotic; 78% female; 9 to 30 years old) when they visited the University of Hong Kong-Shenzhen Hospital to undergo X-ray examinations in June to August 2020. Any subject with other conditions, such as physical, neurological, or mental disorders, that would affect their back muscles was excluded. The youngest participant was 9 years old, and the oldest participant was 30 years old. Their basic information is listed in Table 1. The experiment was approved by the Human Participants Ethics Sub-Committee of the Hong Kong Polytechnic University (Reference Number: HSEARS20201114001). Written informed consent was obtained from all of the participants or their parents/guardians after they were provided details of the study both orally and in the written format.

### 2.2. Experimental Design

**Figure 1:**
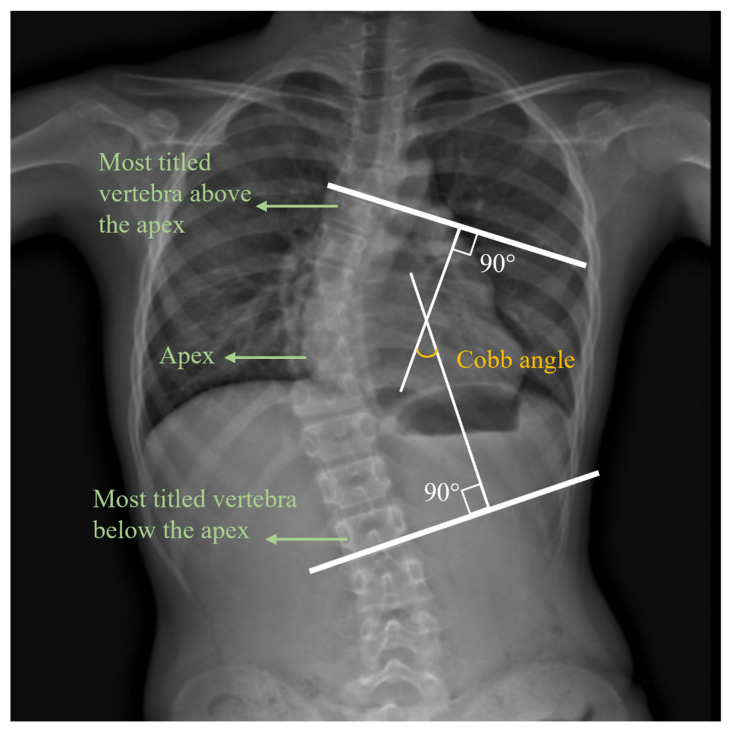
An X-ray image of the Cobb angle.

Prior to the EMG component of the study, all of the participants underwent a clinical neurological examination and radiological assessment of their coronal Cobb angle in standing position, which is shown in Table 1. The measurement of the Cobb angle is shown in Figure 1 [30]. The first step was to identify the most tilted vertebra above and below the apex of the curve. Then, the Cobb angle was measured between the tangents of the upper and lower endplates of the upper and lower vertebra, respectively [31]. This measurement was conducted by a physician or physiotherapist.

The equipment used to obtain the sEMG signals is a wireless EMG sensor system (Noraxon USA Inc., Scottsdale, AZ, USA), which can transmit data from the electrodes to a receiver, and thus is a convenient means of obtaining EMG signals in dynamic conditions. This sensor system has a 4000 Hz EMG sampling rate, real-time synchronization, and low baseline noise. The used electrodes are disposable self-adhesive Ag/AgCL snap electrodes (Noraxon USA Inc., Scottsdale, AZ, USA). The myoMUSCLE™ software (Noraxon USA Inc. Scottsdale, AZ, USA) was used for EMG data analysis.

**Figure 2:**
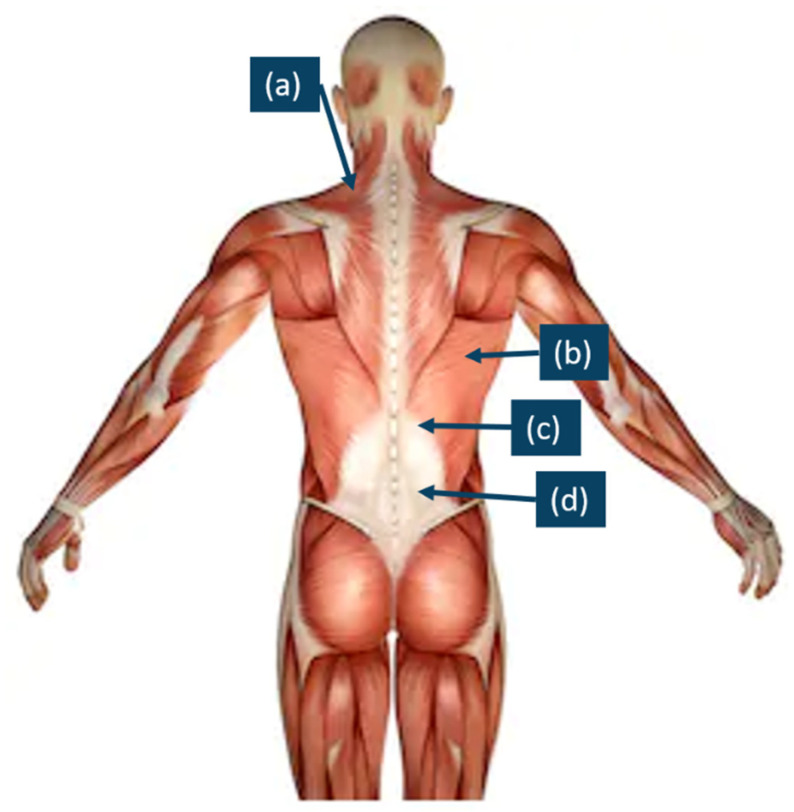
Electrode placement. Upper trapezius (TRAP) (a), latissimus dorsi (b), erector spinae—thoracic (c), and erector spi-nae—lumbar (d).

**Figure 3:**
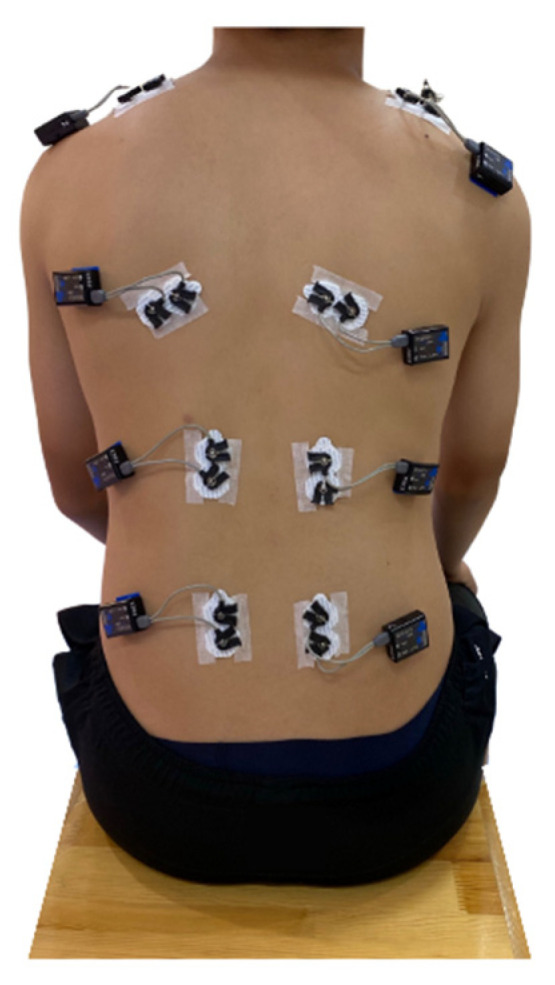
EMG test while sitting (static condition).

**Figure 4:**
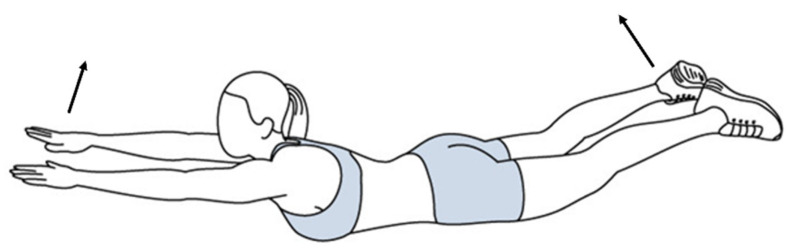
Dynamic pose.

In this study, the experimental protocol included three consecutive steps. The first step was preparation, which was based on the SENIAM instruction. The surface of the skin of the back of the participants was shaved and cleaned with alcohol to remove any body oil or sweat, thus ensuring that the skin impedance was low so that EMG signals could be detected [32]. After that, eight electrodes were attached to the muscle bellies of the upper trapezius (TRAP) (a), latissimus dorsi (b), erector spinae—thoracic (c), and erector spinae—lumbar (d) muscles on both sides, which are shown in Figure 2. The direction of each electrode was in the same direction of the muscle fibers. Following the recommendations of the use of sEMG for the noninvasive assessment of muscle activity, the electrodes for all of the participants were positioned on the surface of the skin by the same researchers in this study [33]. The second step was the participants’ sitting as sitting was a common posture for everyone. The participants were asked to sit on a wood stool with knees and ankles at nearly 90°, and at the same time, the EMG signals were tested (static condition). The hands lay on the lap. The requirements for sitting were to hold still, look straightforward, breathe steadily, and relax (as shown in Figure 3). The duration was 10 s for each participant, which exceeded one respiratory cycle. In the third step, the EMG data were recorded in a dynamic condition. Dynamic movement should require all tested paraspinal muscles. At the beginning, the participants were instructed to calmly lie in a prone position on a mat, with their legs straight and arms outstretched in front. Then, they were to raise both their arms and legs at the same time as high as they could to form a bowl shape, as shown in Figure 4. This pose was repeated six times to ensure that it was performed correctly. Additionally, the holding duration was 3 s each time.

### 2.3. Machine Learning Approaches

Random forests and SVMs are two powerful ML models used for classification, regression, and other functions. SVMs were published by Vapnik and Cortes in the year 1995 [34]. This approach is a robust supervised ML approach that can construct a hyperplane or a set of hyperplanes for an accurate separation. As for the random forest algorithm, it was presented by Breiman in the year 2001 [35]. Its basic idea is that there are several randomized decision trees, and the result is based on the aggregation (majority vote or averaging) of the result for each tree [36]. Previous studies have predicted the likelihood of scoliosis through ML methods based on surface topography methods or gait analysis [37,38]. To investigate the influence of muscle activity on the likelihood of scoliosis, two kinds of classifiers have been developed based on participant information and EMG signals.

**Figure 5:**
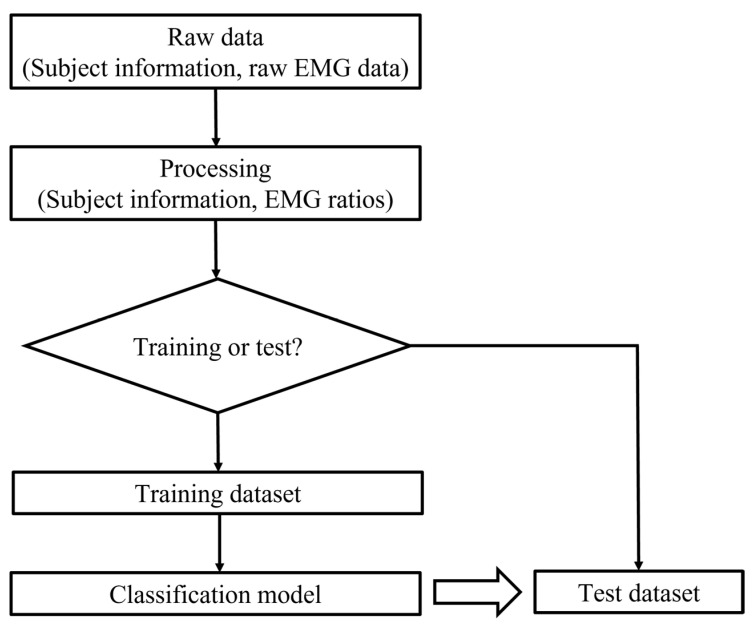
Flow chart of the classification algorithm.

First, raw EMG data had to be processed, which involved filtering and calculating the root mean square (RMS). The window was 250 m, which was suitable for static and dynamic conditions [39]. Two filters were applied, including a band pass filter (10 to 500 HZ) to remove unacceptable artifacts and a notch filter (60 Hz) to eliminate noise [36,40]. The RMS values for all of the collected data were calculated because the RMS value provides the most information on the sEMG signal amplitude as it represents the power of the sEMG signals and can produce analyzable waveforms. Ratios of larger to smaller EMG amplitudes were then calculated by dividing the RMS of one side by the RMS of the other side. The ratios should be equal to or greater than one. Then, feature set data were obtained through the eight EMG ratios and five basic profile variables of the participants (gender, age, height, weight, and angle of trunk rotation (ATR)). The output is 1 or 0, which represents the participants with and without scoliosis, respectively. The classifiers were created by using the Classification Learner app in MATLAB^®^ (version 2017a, MathWorks Inc., Natick, MA, USA). The flow chart of this part of the study is shown in Figure 5. The random forest and SVM classifiers were then compared.

Data from the 106 participants were randomly categorized into two groups: one with 100 participants and another with 6 participants. The first group was used to train the ML models, and the second was used to validate the models. During the training process, cross-validation techniques were used to validate the statistical significance of the classification performance. A five-fold cross validation for these two classifiers was used by splitting the entire dataset into an 80:20 ratio for the training and the validation sets, respectively.

### 2.4. Importance Analysis

An important question in scoliosis classification studies is whether a selected variable is really important and useful for conducting classifications and making predictions. There are many attributes that contribute to scoliosis, such as gender, age, and ratio of the paraspinal muscles. To further understand the feature that has the most influence on the detection of scoliosis, feature importance is calculated by using a random forest model. The advantage of random forests is that there is a validation process, which compares the decision trees. In this paper, 30% of the sample was selected for cross validation in this importance analysis model. The rest was resampled. The basic idea behind using random forests is described as follows:

1. There are about 1/3 data left after the training of each decision tree in the random forest method, called out-of-bag (OOB) data (Breiman, 1996). The OOB data are used to estimate the trained trees and calculate the data error, which is marked as errOOB1 for each decision tree.
2. Noise is added randomly to interfere with the features of all of the OOB samples. This OOB data error is calculated and marked as errOOB2.
3. We assume that there are N trees in total, and the importance of a feature is determined by sum(errOOB2-errOOB1)/N.

In this way, the importance of each feature can be reflected in the OOB data error. If the OOB data error significantly increases when random noise is added, then this feature greatly influences the result, which in this case is the occurrence of scoliosis. In other words, this feature has higher importance. The related calculation process is shown as follows.

Suppose that there are *k* categories, the Gini index can be calculated as:

(1) $$Gini\left(p\right)=\overset{k}{\underset{k=1}{\sum }}p_{k}\left(1-p_{k}\right)=1-\overset{k}{\underset{k=1}{\sum }}p_{k}^{2}$$

where $p_{k}$ is the weight of the k-th category.

On the other hand, for feature j, the Gini index at node m can be calculated by using different Gini index values before and after branching. Assuming that VIMjm denotes the change value of the Gini index of feature j at node m, GIm denotes the Gini index before branching, and GIl and GIr denote two new nodes after branching, then:

(2) $${\mathrm{VIM}}_{\mathrm{jm}}={\mathrm{GI}}_{m}-{\mathrm{GI}}_{l}-{\mathrm{GI}}_{r}$$

If feature j appears M times in the decision tree i, the importance of this feature for the decision tree is:

(3) $${\mathrm{VIM}}_{\mathrm{ij}}=\sum_{m\in M}^{\;}{\mathrm{VIM}}_{\mathrm{jm}}$$

Then, the importance of feature j is:

(4) $${\mathrm{VIM}}_{j}=\frac{1}{n}\sum_{i=1}^{n}{\mathrm{VIM}}_{\mathrm{ij}}$$

where n is the number of decision trees in the random forest model.

## 3. Results

**Figure 6:**
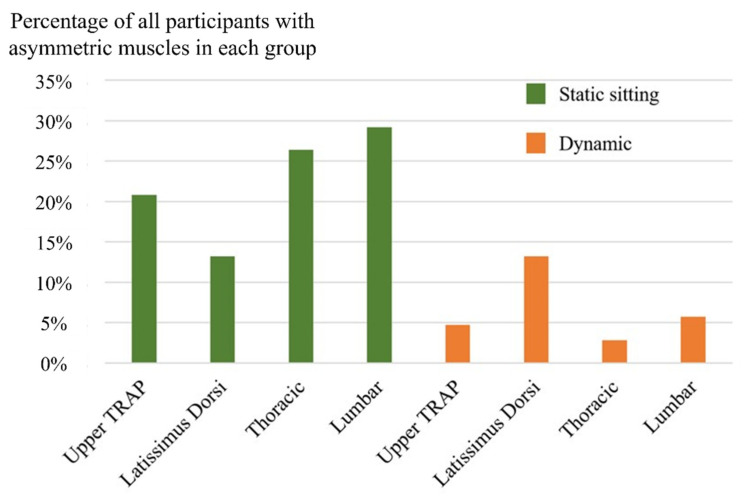
Participants with significantly asymmetric paraspinal muscles.

**Figure 7:**
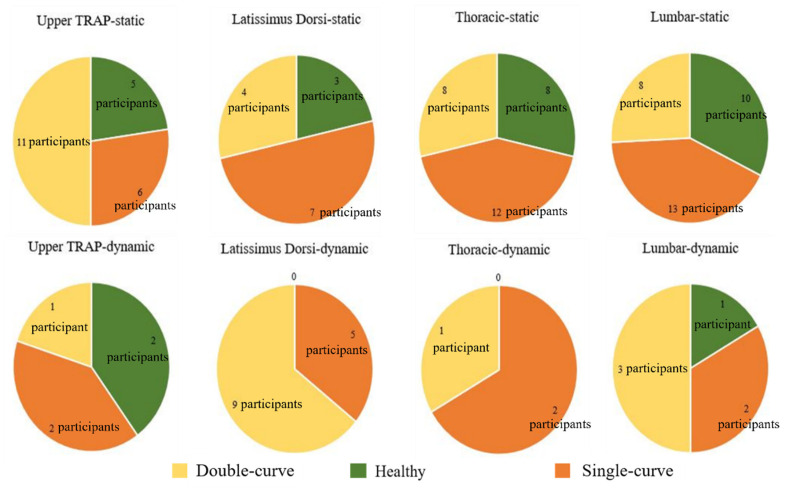
Participants with significant differences between left and right muscles based on group.

First, to investigate the features for the participants with asymmetric paraspinal muscles, the participants with calculated ratios larger than two were grouped together, which is shown in Figure 6. Based on the radiography results, the participants were then divided into three groups—participants without scoliosis, patients with double-curve scoliosis, and patients with single-curve scoliosis. Then the characteristics of each group were observed and analyzed. The number of participants in each group with significant differences between the left and right muscles is plotted in Figure 7. It can be easily observed in the figures that the asymmetry of the muscles, for scoliotic and nonscoliotic participants, is found much more often in the static as opposed to the dynamic condition.

Second, among the results from the two ML approaches, the random forest classifier showed a 73% accuracy with the MATLAB Classification Learner, while the SVM classifier showed a 78% accuracy. The sensitivities were 0.78 and 0.80, respectively, and the specificities were 0.22 and 0.20, respectively. The 95% confidence intervals were also calculated for both classifiers, which were 0.14 and 0.13, respectively. However, their accuracy was 50% when the results were calculated by using the data in the second group. To be specific, three of the six individuals were correctly diagnosed (whether the participant was a scoliosis patient).

**Figure 8:**
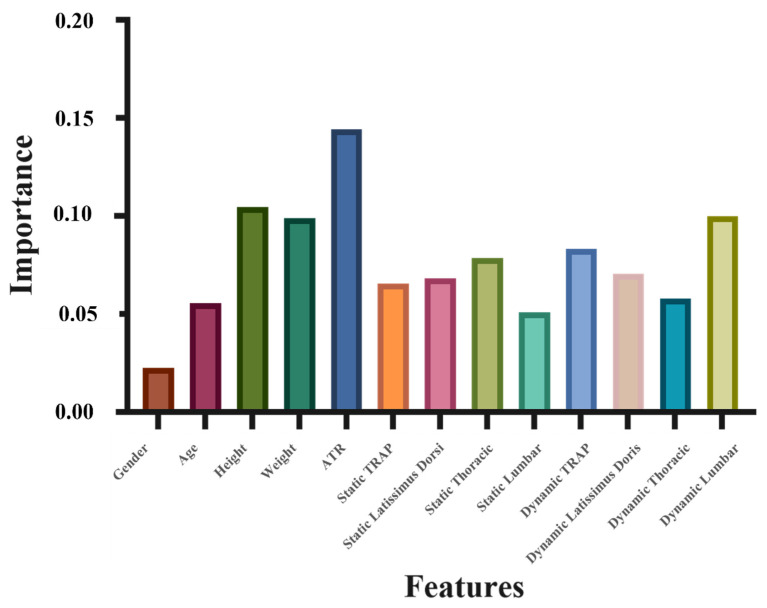
Importance of each feature based on random forests.

Finally, Figure 8 shows the importance of each feature. Thirteen features, which were ranked in the order of importance, were ATR, height, dynamic ratio of erector spinae (lumbar), weight, dynamic ratio of TRAP, static ratio of erector spinae (thoracic), dynamic ratio of latissimus dorsi, static ratio of latissimus dorsi, static ratio of TRAP, dynamic ratio of erector spinae (thoracic), age, static ratio of erector spinae (lumbar), and gender.

## 4. Discussion

The EMG activity of the paraspinal muscles of 106 participants was tested in this study. The participants included subjects with and without scoliosis. In addition, the work was conducted in both static (sitting) and dynamic conditions. First, the characteristics of the participants with very asymmetrical muscles were investigated. A considerable percentage of the participants were accustomed to sitting without balancing their bilateral back muscles evenly, which indicates the difficulty of maintaining a good sitting posture. In addition, Kwok et al. [41] concluded that sEMG can be used to conduct biofeedback training for patients with scoliosis. During the training, the postures of the patients were adjusted to achieve a more balanced sEMG ratio for the TRAP, latissimus dorsi, erector spinae—thoracic, and erector spinae—lumbar regions between two sides. Regular training sessions could help them to develop a more balanced posture. Thus, biofeedback training with the use of sEMG could be a viable method to instilling a balanced sitting or standing habit. Another notable observation in Figure 7 is that even though the percentage of the participants without scoliosis was smaller, there were still some participants without scoliosis who had very imbalanced paraspinal muscles.

Regarding detection of scoliosis with the use of ML approaches, the accuracies of the random forest and SVM classifiers were 73% and 78%, respectively, both of which are not ideal. The reasons the accuracies were somewhat lacking were: (1) the sample size of 100 participants was not large enough to build this kind of classifier [42], and (2) the set of profile variables used in this study (age, gender, height, weight, ATR, and EMG ratios) did not have a strong-enough correlation with the presence of scoliosis. Therefore, the most important eight features (ATR, height, dynamic ratio of erector spinae (lumbar), weight, dynamic ratio of TRAP, static ratio of erector spinae (thoracic), dynamic ratio of latissimus dorsi, static ratio of latissimus dorsi) were selected for building new ML models to remove the features with low correlation and improve the accuracy. Then, the random forest classifier showed a 77% accuracy, while the SVM classifier showed a surprising 85% accuracy. The superior performance of the SVM classifier makes it suitable for this study.

Subsequently, an importance analysis was carried out to determine the variables that are highly correlated to pathogenesis by using a random forest model. In Figure 8, feature 5, or the ATR, has the most important role in predicting scoliosis, which agrees with previous findings in the literature [43]. Measuring the ATR is now a reliable and reproducible method for scoliosis screening [44]. It can also be observed that height is second in importance, and weight ranks fourth in the importance analysis for scoliosis prediction. Parents and doctors need to heed the abnormalities of the height or weight values for children due to their significance. The standards are found in the study of Tanner and Whitehouse [45]. The most interesting finding is that the dynamic EMG ratios of the erector spinae (lumbar) muscles are also very important (third in importance). A widely accepted fact is that the erector spinae muscle group is the most important group of muscles for the stability of the spine [46]. Moreover, Cheung et al. [19] indicated that larger EMG ratios can be found at the lower end vertebra of young patients with a progressive spinal curvature. Hence, the symmetry of the left and right erector spinae (lumbar) muscle activity during movement can be used as supplementary information for the diagnosis of scoliosis.

## 5. Conclusions

This paper presented a cross-sectional research to study the characteristics of participants with imbalanced muscle activity and the relationships among 13 features (physical features and EMG signal value). The results showed that muscle activities were more easily imbalanced during sitting than during movement. Moreover, importance analysis ranked 13 features, which were ATR, height, dynamic ratio of erector spinae (lumbar), weight, dynamic ratio of TRAP, static ratio of erector spinae (thoracic), dynamic ratio of latissimus dorsi, static ratio of latissimus dorsi, static ratio of TRAP, dynamic ratio of erector spinae (thoracic), age, static ratio of erector spinae (lumbar), and gender, in descending order by correlation. According to it, a new SVM classifier using the most important 8 features showed good accuracy (85%).

## Figures and Tables

**Table 1:** Participant information.

		No. of Participants	No. of Male Participants	No. of Female Participants	Age/Years Old	Height/m	Weight/kg	ATR/°	Cobb Angle/°
Participants with scoliosis	Single-curve scoliosis	39	12	27	14.51 ± 3.34	1.62 ± 0.09	46.68 ± 11.39	6.74 ± 4.35	21.97 ± 10.11
	Double-curve scoliosis	34	2	32	15.00 ± 4.51	1.62 ± 0.07	47.04 ± 9.27	8.50 ± 3.61	27.59 ± 9.94
	All	73	14	59	14.63 ± 3.82	1.61 ± 0.09	46.55 ± 10.46	7.35 ± 4.12	23.72 ± 10.65
Participants without scoliosis		33	10	23	14.36 ± 4.78	1.57 ± 0.12	44.85 ± 10.14	2.79 ± 3.23	1.03 ± 2.03
All		106	24	82	14.44 ± 4.11	1.60 ± 1.01	45.68 ± 10.45	6.12 ± 4.38	17.48 ± 13.61
